# Understanding group A streptococcal pharyngitis and skin infections as causes of rheumatic fever: protocol for a prospective disease incidence study

**DOI:** 10.1186/s12879-019-4126-9

**Published:** 2019-07-17

**Authors:** Julie Bennett, Nicole J. Moreland, Jane Oliver, Julian Crane, Deborah A. Williamson, Dianne Sika-Paotonu, Matire Harwood, Arlo Upton, Susan Smith, Jonathan Carapetis, Michael G. Baker

**Affiliations:** 1 Department of Public Health, University of Otago, Wellington, New Zealand; 2 School of Medical Sciences, University of Auckland, Auckland, New Zealand; 3 Doherty Institute, University of Melbourne, Melbourne, Australia; 4 Department of Medicine, University of Otago, Wellington, New Zealand; 5 Deans Department and Department of Pathology and Molecular Medicine, University of Otago, Wellington, New Zealand; 6 Wesfarmers Centre for Vaccines and Infectious Diseases, Telethon Kids Institute, Perth, Australia; 7 Faculty of Health, Victoria University of Wellington, Wellington, New Zealand; 8 Faculty of Medical and Health Sciences, University of Auckland, Auckland, New Zealand; 9 Southern Community Laboratory, Dunedin, New Zealand; 10 Labtests, Auckland, New Zealand; 11 Telethon Kids Institute, University ofWestern Australia, Perth, Australia

**Keywords:** Group a streptococcus, Acute rheumatic fever, Rheumatic heart disease, Sore throat, Skin infection, *S. pyogenes*, Pharyngitis, Children, Strep throat, Impetigo, GAS

## Abstract

**Background:**

Group A Streptococcal (GAS) infections cause the autoimmune disease acute rheumatic fever (ARF), which can progress to chronic rheumatic heart disease (RHD). Treating pharyngitis caused by GAS with antibiotics is important in preventing ARF. However, it is difficult to distinguish these infections from GAS carriers. There is growing evidence for GAS skin infections as a cause of ARF. This study will identify the incidence of true GAS pharyngitis and serological responses to GAS skin infections. The effectiveness of antibiotics for these conditions will be explored, and modifiable risk factors. Serum antibody titres indicating the upper limits of normal (ULN for ASO/ADB antibodies) will be established alongside carriage rates in asymptomatic children.

**Methods:**

This is a prospective disease incidence study, with an associated case-control study. The study population includes 1000 children (5–14 years) from Auckland, New Zealand, 800 of whom have visited their healthcare professional, resulting in a throat or skin swab for GAS*,* and 200 who are asymptomatic. The conditions of interest are GAS throat swab positive pharyngitis (*n* = 200); GAS carriage (*n* = 200); GAS negative throat swab (*n* = 200); GAS skin infections (*n* = 200); and asymptomatic controls (*n* = 200). All participants, except asymptomatic controls, will have acute and convalescent serological testing for ASO/ADB titres (collected < 9 days, and 2–4 weeks following symptom onset, respectively), alongside viral PCR from throat swabs. Asymptomatic controls will have ASO/ADB titres measured in one blood specimen and a throat swab for microbial culture. Caregivers of children will be interviewed using a questionnaire and any GAS isolates identified will be *emm* typed. The persistence of GAS antibodies will also be investigated.

**Discussion:**

Findings from this study will fill critical gaps in scientific knowledge to better understand the pathophysiology of ARF, improve clinical management of GAS infections, and design more effective ARF prevention programmes. In particular it will measure the incidence of true, serologically confirmed GAS pharyngitis; assess the immune response to GAS skin infections and its role as a cause of ARF; examine the effectiveness of oral antibiotics for treating GAS pharyngitis and carriage; and identify whether risk factors for GAS infections might provide intervention points for reducing ARF.

## Background

### Rheumatic fever and rheumatic heart disease

Group A Streptococcus (GAS) causes a broad spectrum of diseases, from superficial infections such as pharyngitis and impetigo, to invasive infections like necrotizing fasciitis and streptococcal toxic shock syndrome. GAS infections can also lead to the development of autoimmune diseases, such as acute rheumatic fever (ARF) [1, 2]. In an estimated 60% of ARF cases, carditis progresses to chronic rheumatic heart disease (RHD), which can produce permanent heart valve damage [3]. Unless further episodes of ARF are prevented with intramuscular injections of benzathine penicillin G every 28 days, ARF patients are likely to suffer worsening cardiac damage and increasing chances of heart failure, stroke and early death [4]. Globally approximately 34 million people are affected by RHD with an additional 47 million having asymptomatic damage to their heart values [5, 6]. Annually, severe GAS infections are responsible for an estimated 517, 000 deaths [3].

ARF was once common throughout the Western world, with entire hospital wards dedicated to caring for patients. Incidence rates declined sharply over the twentieth century, coinciding with improvements in living conditions and later the widespread use of antibiotics to treat streptococcal infections [7, 8]. Despite a world-wide decline, ARF and RHD remain important causes of morbidity and preventable early death in the low- and middle-income countries [3, 9]. ARF has also persisted in indigenous communities in some developed countries. In New Zealand, there are large and widening ethnic disparities in ARF rates [10, 11]. Ethic inequalities are particularly pronounced amongst Indigenous Māori and Pacific population groups living in New Zealand, with more than nine out of every 10 cases occurring in these groups [12]. The rate of new cases of ARF notified in New Zealand from July 2017 to June 2018 for Māori children aged 5–12-years was 26 per 100,000 and for Pacific children aged 5–12-years was 94 per 100,000, and for European/Other children was < 1 per 100,000 [12].

ARF largely occurs in children, particularly those living in socioeconomic deprivation. From July 2015 to June 2018, 64% of all new ARF cases in New Zealand were children aged 5–14-years [11], and between 2010 and 2013 people living in the most deprived areas of New Zealand were found to be 33 times more likely to develop ARF compared with those in the least deprived areas. Children living in the most deprived socioeconomic quintile have a 1:150 chance of being hospitalised with ARF by the time they reach the age of 15 years [9].

In response to the high rates of ARF and increasing ethnic inequalities, the New Zealand Ministry of Health introduced the Rheumatic Fever Prevention Programme (RFPP) in 2011. The RFPP placed a strong emphasis on sore throat management – that is, prompt detection by culturing throat swabs and if the swab was positive for GAS, a course of broad-spectrum antibiotics was provided to treat the infection promptly with the aim of preventing subsequent ARF [13, 14].

### Incidence of true group A streptococcal pharyngitis

Treating pharyngitis caused by GAS infection with antibiotics is an important measure for preventing ARF. A major challenge to this approach is that it is extremely difficult to distinguish patients with true GAS pharyngitis from carriers who have viral pharyngitis when throat swab culturing alone is used to diagnose GAS pharyngitis [15, 16]. To date most studies investigating the incidence of GAS pharyngitis have identified cases using throat swabbing and culture. In some cases, a rapid antigen detection test (RADT) is used; however, laboratory culture is still considered the most specific means of GAS confirmation [17, 18]. Clinical symptoms are relatively non-specific so are often used in combination with RADT or culturing when diagnosing GAS pharyngitis [19–21]. At present, the ‘gold standard’ method for diagnosis of true GAS pharyngitis utilises serological testing. This method measures the titres of antistreptolysin (ASO) and anti-DNase B (ADB) antibodies in two blood samples taken two-to-four-weeks apart [22] with seroconversion thought to distinguish true GAS pharyngitis from pharyngeal GAS carriers.

Only a small number of studies have investigated the incidence of serologically confirmed GAS pharyngitis. A recent meta-analysis assessed the prevalence of GAS pharyngitis and carriage in different settings [16]. The analysis identified 285 eligible studies and reported that in clinical settings; approximately 10% of children swabbed with a sore throat had serologically-confirmed GAS pharyngitis, with this increasing to around 50–60% when the child was GAS culture-positive. The analysis reported a prevalence of 10.3% (6.6–15.7%) for serologically-confirmed GAS pharyngitis in children from high-income countries and an asymptomatic GAS carriage prevalence of 10.5% (8.4–12.9%). Lower carriage prevalence was detected in children from low/middle income countries (5.9%, 4.3–8.1%) [16].

As most sore throats are caused by viral infection, it is likely that many patients considered to have pharyngitis caused by GAS are in fact GAS carriers with concurrent viral pharyngitis [23–25]. Such individuals often receive false positive diagnoses and unnecessary antibiotic treatment, with carriers unlikely to benefit from antibiotic treatment. The Infectious Diseases Society of America makes a strong recommendation against routine antibiotic treatment of carriers [26]. Identifying the incidence of true (serologically confirmed) GAS pharyngitis is of critical importance when seeking to guide treatment, target interventions and inform vaccine development [23, 27].

### Group A streptococcal skin infections

There is growing evidence regarding the potential importance of GAS skin infections as a cause of ARF, either alone or in combination with GAS pharyngitis [1, 2, 28–30]. In New Zealand rates of skin infections are markedly higher in New Zealand Māori and Pacific peoples compared with the New Zealand European/Other population groups [29]. Recent figures show that Māori and Pacific children under 9 years of age suffer disproportionately high rates of GAS skin infections in comparison to other ethnicities [31]. One New Zealand study conducted in the Tairawhiti region estimated an annual incidence rate of 106.7 cases per 1000 children; i.e. approximately one in nine children in the study region consulting their doctor for a skin infection each year [32]. Among skin infection cases hospitalised in that region, GAS was the second most isolated organism with 31% of children who had a microbiological swab taken positive for GAS; suggesting that this organism is a major contributor to such infections at a population level [33].

### Risk factors for group A streptococcal infections

Identifying potentially modifiable risk factors for relevant GAS infections (pharyngitis and/or skin infections) is critical for the design of evidence-based ARF programmes. There is little published research in this area. One Australian study that has investigated risk factors for serologically confirmed GAS pharyngitis did not detect any association with crowding, gender, socio-economic status, or time spent at preschool, childcare or school [23]. Participating families were mainly small to moderate-sized and middle-class, so this study was not well powered to measure the effects of socio-economic deprivation [23], and therefore findings cannot be extrapolated to the groups at most risk of developing ARF in New Zealand.

### Risk factors for rheumatic fever

The literature base of studies on risk factors for ARF and RHD is much larger than it is for GAS infections, though the studies are generally considered to be of poor quality [34]. One systematic literature review concluded that the findings supported a causal association between household crowding and low socioeconomic position and the development of ARF [34]. In New Zealand an ecological study found that the risk of ARF was associated with neighbourhood deprivation, household crowding, and the proportion of 5–14-year-old children living in the area [35].

### Aims and research questions

The purpose of this research work is to fill critical gaps in scientific knowledge that are needed to support New Zealand’s Rheumatic Fever Prevention Programme (RFPP). This research will also add to our understanding of the pathophysiology of ARF. New Zealand invested more than $65 million in the RFPP, with most of these resources used to support large-scale sore throat management interventions. Unfortunately, we do not know what proportion of children treated through the RFPP actually had true GAS pharyngitis and much of the associated antibiotic use may have been unnecessary.

Similarly, we know little about the role of GAS skin infections, which may be an important precipitating event for ARF and thus might provide a key target for interventions. This study will also investigate the risk factors associated with GAS pharyngitis, carriage and skin infections, thus providing knowledge about potential prevention measures. The specific study aims are to:

1. Determine the incidence and distribution of GAS pharyngitis (serologically confirmed) amongst children presenting to their health care professional with a sore throat.
2. Determine the incidence of GAS positive skin infections amongst children presenting to their health care professional with a skin infection and examine the serological response to these infections.
3. Measure the prevalence of GAS carriage in asymptomatic children, aged 5–14 years.
4. Compare the genomic epidemiology (including *emm*-typing) of GAS associated with pharyngitis and skin infections to those associated with the GAS carrier state, ARF and healthy control participants, as well as isolates detected in throat swabs obtained from GAS skin infection participants.
5. Identify epidemiological risk factors for GAS pharyngitis and skin infections, and assess their potential as modifiable risk factors for these infections and ARF.
6. Assess the contribution of viral infection to presumed GAS pharyngitis seen in primary care.
7. Measure the effectiveness of antibiotic treatment on pharyngeal GAS carriage compared with pharyngitis.
8. Measure the persistence of GAS antibodies up to 6 months following the initial sample collection.
9. Determine age-specific upper limit of normal (ULN) values for ASO and ADB titres in children aged 5–14 years.

## Methods

### Study design and population

The study design is a prospective disease incidence study that has an associated case-control study. The conditions of interest are divided into the following groups:

1. GAS pharyngitis cases (*n* = 200 participants),
2. GAS carriers (*n* = 200 participants),
3. GAS negative sore throat cases (*n* = 200 participants),
4. GAS skin infections (*n* = 200 participants),
5. Asymptomatic controls (*n* = 200 participants).

The study aims to include 1000 school aged (5–14-year-old) children in Auckland, 800 of whom have visited their healthcare professional, with the result of a throat or skin swab being sent to Labtests laboratory (sole diagnostic community laboratory service provider for the Auckland region) for investigation of presumed GAS infection. Families of children with a GAS positive throat or skin swab, or a GAS negative throat swab, will be phoned by Labtest’s microbiology staff and invited to take part in the study. In addition, 200 asymptomatic children who have previously taken part in the New Zealand Health Survey and consented to being contacted for further research will be recruited. CBG Health Research Limited, who provides independent public sector research services in New Zealand, will contact all Labtest referrals and potential asymptomatic participants (identified from previous participants in the New Zealand Health Survey) to arrange an initial home visit and specimen collection.

**Fig. 1:**
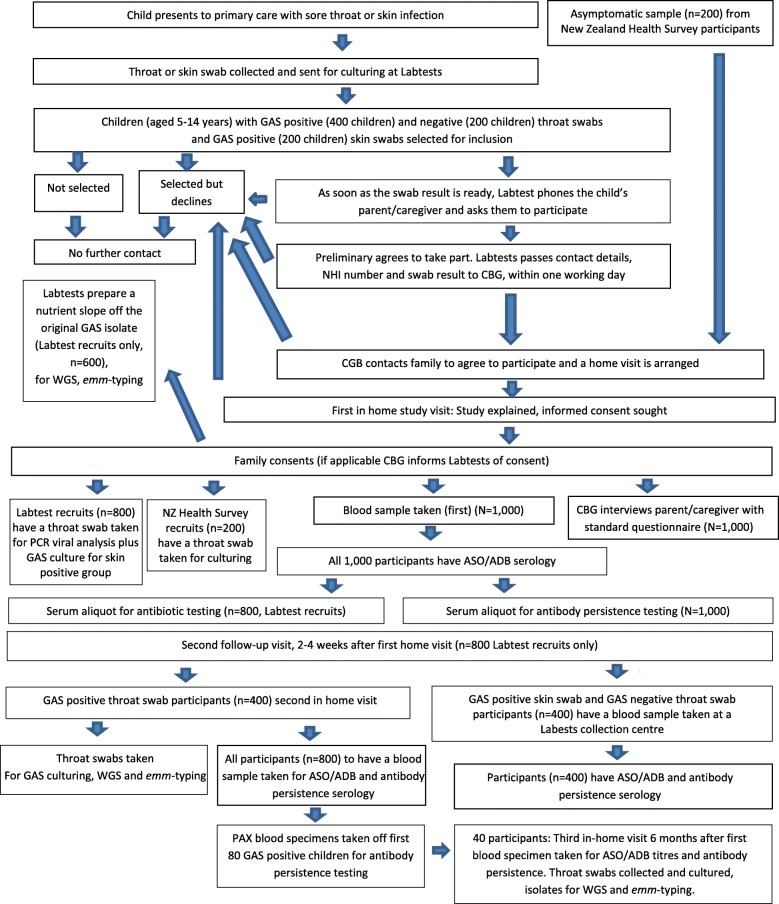
Flow chart of recruitment, specimen collection and data gathering

Figure 1 outlines the study recruitment, data gathering, specimen collection and laboratory analyses. Participants with the first four conditions of interest (*n* = 800) will have acute and convalescent serological testing for ASO/ADB titres (taken < 9 days post swab collection and 2–4 weeks post-acute blood specimen collection, respectively). Asymptomatic participants in the fifth group (*n* = 200) will have one blood specimen taken for ASO/ADB titre assays and one throat swab for microbiological culturing. Identification of GAS negative sore throat cases will be determined by routine culturing. Unequivocal GAS pharyngitis cases will be those participants who present with a GAS positive throat culture and a > 0.2 log-10 rise in ASO or ADB antibody titres between their acute and convalescent serum samples as previously described [23]. Probable GAS pharyngitis cases will include any participant where the first serum sample is taken after the acute phase, with ASO and/or ADB titres that are above the ULN determined for this study. Carriers will be determined by routine swab culturing that yields GAS but no increase in ASO or ADB titres, nor titres above the ULN. Skin infection cases will be determined using routine culturing of skin swabs that yield GAS.

### Inclusion criteria

The study aims to recruit approximately equal numbers of New Zealand Māori, Pacific and non-Māori/non-Pacific children, aged between 5 and 14 years, over an 18-month period. Recruitment will include all areas of Auckland, which consists of three separate district health boards (DHBs); Waitemata, Auckland and Counties Manukau. Timing of the first blood sample (*n* = 800 participants) must be within 9 days of symptom onset and preferably within 7 days.

### Consent

A CBG researcher will visit the caregiver/parent and child in their home to explain the study. The researcher will provide the parent/caregiver and the child with a participant information sheet and if needed the researcher will read/explain this sheet. Information and consent sheets will be translated from English into Te Reo Māori, Cook Island Māori, Samoan, Tongan and Niuean. If language difficulties are encountered during the consent process, language translation services will be used.

### Data collection

#### Interviews

To identify potentially modifiable risk factors at the first home visit all parents/caregivers of participants will be interviewed, using a questionnaire designed for the Rheumatic Fever Risk Factors Study (JVP 13/959). This questionnaire draws on other pre-existing questionnaires where appropriate to maximise comparability. Questions used in the New Zealand Health Survey [36], SHIVERS study [37], and a recent survey of housing conditions in hospitalised children [38] were also used.

The interviews will be carried out by experienced researchers, specifically trained for this study. The interviewer will type responses directly into a laptop computer using the ‘Survey System’ Computer Assisted Personal Interview (CAPI) software. Showcards with predetermined response categories and pictorial examples will be used where required to assist respondents. There will be no ‘rule’ regarding who is present during the interview, and as such multiple family members may be present (and thus may assist the child/caregiver with answering questions).

### Specimen collection

Initial throat and skin wound cultures will be collected by participants’ healthcare providers. Trained CBG researchers will take the acute blood sample and throat swabs at the first home visit for all participants (*N* = 1000).

Those participants who had an original positive GAS throat swab (*n* = 400) will receive a second home visitation, 2–4 weeks after the first home visit, where the CBG researcher will take a convalescent blood sample and a throat swab. Participants whose initial throat swab was GAS negative, and participants who had a positive GAS skin swab, do not require a throat swab to be collected and so will visit a Labtests collection centre, 2–4 weeks after their home visit, to have their second convalescent blood sample taken. An additional sample of *n* = 40 GAS throat swab positive cases with seroconversion will receive a third home visit from CBG staff, 6 months after study enrolment where a blood sample and a throat swab will be collected to investigate the persistence GAS antibody responses.

### Specimen testing

#### GAS culture

All GAS culture will be carried out by Labtests, Auckland using standard laboratory methods. GAS cultures will be quantified to assess the density of bacterial growth and scored accordingly using 0 (no growth) – 3 (heavy growth) categories.

#### ASO/ADB titres and antibody specificity, function and persistence

ASO/ADB titres will also be determined by Labtests using standard methods. Un-used serum will be aliquoted into vials and frozen at − 80 °C at Labtests for future research on antibody responses to GAS at the University of Auckland. A sub-study of participants (*n* = 40) will be followed up to investigate GAS antibody persistence including examination of the expressed antibody repertoire.

Sera will be tested for the presence of antibodies specific for the infecting strain type by ELISA against M-protein peptides derived from the hypervariable region of the protein as previously published [39]. The function of these type specific antibodies will be tested in recently developed opsonophagocytic assays that measure bactericidal activity [40]. Finally, the expressed antibody repertoire will be studied using B-cell receptor sequencing methods [41] to gain insight into B-cell repertoire structure following GAS infection.

#### Respiratory pathogen polymerase chain reaction (PCR)

CBG researchers will use Copan FLOQ swabs and universal transport media to collect a throat swab from all Labtest recruits (*n* = 800) at the initial home visit. These swabs will be tested for viral pharyngitis, using PCR to detect one or more viral pathogens. Viral PCR analysis will be carried out at Southern Community Laboratories in Dunedin, New Zealand. The respiratory panel tests include: Influenza A, Flu typing, Influenza B, Human metapneymovirus, Adenovirus group B C and E, Human parainfluenza virus 1, 2, 3 and 4, Rhinovirus and enterovirus, human Respiratory Syncytial virus. The following bacteria will also be identified; Mycoplasma pneumoniae, Bordetella pertussis, Bordetella spp. (IS481).

#### Whole genome sequencing (WGS) and in silico *emm*-typing

All GAS isolates from the initial culture requested by the healthcare professional, will be sent to the Microbiological Diagnostic Unit Public Health Laboratory (MDU PHL), Doherty Institute, University of Melbourne. Isolates will be batched and sent monthly. Genomic DNA will be extracted from single colonies using a QIAsymphony™ DSP DNA Mini Kit (Qiagen) according to manufacturer’s instructions, and WGS will be performed on an Illumina NextSeq 500 platform with 150 bp paired-end reads. An in silico typing tool for rapid determination of *emm*-types from WGS data has been developed at MDU PHL [42], thereby facilitating comparison of data obtained from this study with previous studies using conventional *emm-*typing. Phylogenetic analysis will be performed using the [43] ‘Nullarbor’ pipeline.

GAS skin positive participants will have an additional throat swab collected at the first home visit and GAS throat positive participants (pharyngitis and carriers), will have an additional throat swab collected at the second home visit. These swabs will be cultured, and any GAS isolates will undergo WGS and *emm*-typing, as described.

#### Antibiotic testing

The first four categories of interest (*n* = 800) will have the acute serum specimen tested for antibiotic levels at the Telethon Kids Institute in Perth, Australia. This, in combination with a questionnaire (which asks about antibiotic usage), will investigate antibiotic compliance. Pharmacokinetic characteristics of the participants’ antibiotic metabolism will also be explored.

### Specimen storage

All specimens will be stored for a period of 10 years. The exact location of specimens (including details such as the freezer used for storage), will be recorded on an online track-and-trace database and eventually at a central location at the University of Otago, Wellington.

### Steering committees and ethical review

The study has both a Māori and Pacific Steering Group each comprised of a cross-section of key stakeholders, including researchers, public health workers, clinicians and community representatives (members are listed in the acknowledgements section). These groups reviewed the study questionnaire and protocol to ensure it was safe and appropriate. They also provide advice on cultural specific issues as arise during the study, and will be involved in interpretation and dissemination of research findings. One of the investigators (MH) is an active general practitioner in Auckland. The Study Manager is in ongoing communication with Primary Health Organisations in Auckland. These relationships ensure that the study can receive and incorporate feedback about the study from General Practitioners, nurses and other health care providers.

The Ministry of Health’s Northern-A Health and Disability Ethics Committee (HDEC) approved this study (reference /NTA/262). The Ministry of Health approved the study using participants from the New Zealand Health Survey who had consented to taking part in extra research.

### Study size and power

A systematic review of GAS pharyngitis and pharyngeal carriage reported a pooled prevalence of serologically confirmed GAS infection amongst those with GAS positive throat swabs, of around 50%, for children recruited passively (i.e. where patients present to primary healthcare with a sore throat) [16]. The included GAS pharyngitis studies were based on demonstrating an ASO or ADB antibody titre rise of at least 0.2 log between acute and convalescent serum samples. Based on data from Gisborne, we could expect skin infection consultations at a doctors’ practice to be 107/1000/year [32] and GAS as the causative organisation in 31% [33] implying up to approximately 270 GAS skin infections a year within this population group.

For exposures that are relatively common, such as household crowding (58% in this population) [44], this study size will be able to detect an odds ratios (OR) for disease of 1.8 in exposed subjects relative to unexposed subjects with probability (power) of 0.8 and type I error probability of 0.05 using an uncorrected chi-squared statistic to evaluate the null hypothesis. Table 1 indicates the minimum protective and harmful effects we will be able to distinguish with 80% power when a factor of interest has certain prevalence difference in the control population compared with cases.

**Table 1:** Minimum protective and harmful effects at 80% power

Assumed prevalence of factor in controls (%)	Min. Protective OR detectable	Min. Harmful OR detectable	Power
90	0.430	4.016	0.8
80	0.512	2.358	0.8
70	0.546	2.005	0.8
60	0.558	1.864	0.8
50	0.555	1.803	0.8
40	0.537	1.793	0.8
30	0.499	1.832	0.8
20	0.424	1.954	0.8
10	0.249	2.327	0.8

### Data analysis

The goal of the data analysis is to support effective investigation of the aims and research questions. We will calculate the throat swabbing rate and GAS positive rate for our study population using Labtests laboratory data as the numerator and census population data as the denominator (as we have previously done for several regions of NZ) [45]. Since Labtests conducts all the testing for this population using standardised and consistent methods, this calculation should provide robust population rates. We will then apply the results of serological testing generated by this study (i.e. the proportion of GAS positive individuals that were carriers and those with pharyngitis), to estimate the prevalence/incidence of these conditions at a population level. We will then repeat these analyses for age strata and ethnic groups.

Our results will also allow us to estimate the test performance of GAS culture for detecting GAS pharyngitis in this population and context. We will use GAS serology as the gold standard to assess sensitivity, specificity, positive and negative predictive values of a GAS positive throat culture for detecting GAS pharyngitis.

A separate analysis will compare the distribution of *emm-*types in the study populations (GAS pharyngitis cases, GAS carriers and GAS positive skin infections) to those detected in the Rheumatic Fever Risk Factors Study (ARF cases and healthy controls) and those held at the Institute of Environmental Research (ESR), which are collected as part of national surveillance for ARF, invasive GAS infections and as part of the RFPP in other parts of New Zealand.

Initial univariate analysis will compare the odds of exposure to each risk factor investigated for GAS pharyngitis and GAS skin infection cases compared with our various sets of control/comparison groups, with suitable statistical testing (for example Chi-square test for categorical variables). Multiple logistic regression will be used to calculate adjusted odds ratios. Non-additive interaction theoretically plausible or empirically supported interactions(s) between risk factors will be evaluated. At the end of these analyses, this study will be able to identify, with a reasonable degree of certainty, whether the risk of GAS pharyngitis and GAS skin infection in New Zealand is associated with a number of important risk factors that are potentially modifiable: structural and functional household crowding and bed sharing; poor indoor environments (cold, damp, mould) and fuel poverty; tobacco smoke exposure; limited resources for personal hygiene (e.g. washing, teeth cleaning); poor access to health services, including pharyngitis and skin infection treatment; and poor oral health.

This analysis will begin by describing the distribution of detected viral pathogens across all the study populations of children presenting with sore throats (*n* = 600). It will then focus on comparing the prevalence between the GAS negatives, GAS carriers and GAS pharyngitis groups. Our hypothesis is that virus detection will be relatively common in both the GAS negatives and the GAS carriers (as we expect viral infections to be a frequent reason for symptoms in both groups) and uncommon in the GAS pharyngitis group. The extent of overlap of viral pharyngitis with these syndromes (GAS pharyngitis and GAS carriers) is currently unknown so will be an important and novel finding from this research.

This analysis will assess whether oral antibiotic treatment is as effective at clearing GAS carriage as well as GAS pharyngitis. This comparison will be based on re-swabbing all subjects who are GAS throat-swab positive at the second home visit. This is a clinically important and unanswered question in the field of GAS pharyngitis treatment.

Using ASO/ADB titres from asymptomatic children we will determine age-specific ULN values for children in New Zealand as well as carriage rates among asymptomatic children.

### Dissemination

Multiple methods will be used to disseminate study findings. Findings will be communicated through the peer-reviewed literature and conference presentations with associated media coverage helping inform the wider public. At the conclusion of our study, our results will be made available as both printed and online resources.

We will disseminate our research findings in a clear, concise, and culturally-safe manner with Māori and Pacific communities. For example, we will develop press releases and provide these to Māori and Pacific media. We will participate in meetings and workshops which are relevant to the current study. This process will draw on the expertise of the Māori and Pacific Steering Groups. Members of the research group are already providing advice to the Ministry of Health and Health Promotion Agency of aspects of the RFPP programme and are recognised opinion leaders in clinical aspects of ARF and RHD management.

## Discussion

This study will fill critical gaps in scientific knowledge that are needed to better understand the pathophysiology and risk factors for ARF; improve clinical management of cases; and design more effective ARF prevention programmes. Basic knowledge gaps addressed by this study include the immune response in throat and skin infection which has implications for understanding the potential role of skin infections as initiators of ARF. The study will allow comparison of the GAS *emm*-types associated with GAS pharyngitis and skin infections to those associated with the GAS throat carrier state, ARF, and well controls which may provide insights into GAS reservoirs and sources of infection. It is specifically designed to assess whether risk factors for ARF are likely to be mediated through the risk of GAS infections of the throat and skin.

Findings from this study could contribute to improved clinical management of GAS infections in a number of ways. The ULN (ASO/ADB titres) for at-risk New Zealand children will be determined as part of this study using specimens collected from healthy asymptomatic controls (New Zealand Health Survey sample) and previously collected from healthy controls in the Rheumatic Fever Risk Factors Study. It is recommended that each population establish their own ULN for streptococcal serology due to epidemiological differences in GAS between populations [46]. Findings will provide clinicians with a clearer understanding of the proportion of GAS positive sore throats in at-risk populations that are likely to be serologically confirmed pharyngitis compared with those that are likely carriage. It will specifically identify the proportion that are likely to be associated with viral pharyngitis and whether these proportions vary in a seasonal way.

The largest contribution of this study is likely to be to inform the improved design of rheumatic fever prevention programmes within New Zealand and internationally. Key aspects include whether skin infections need greater emphasis, the effectiveness of oral antibiotic treatment in treating GAS pharyngitis and carriage, and whether there are risk factors for GAS infections that might provide intervention points for reducing the risk of ARF. This study will provide an improved understanding of the immune response in these infections and its duration which can contribute to efforts to develop an effective group A streptococcal vaccine.

The study has limitations, including the representativeness of the study populations recruited and the constraints of the laboratory test methods. The study is based on children who access a healthcare provider when they have a sore throat or skin infection so may not fully represent disease distribution in the population. In particular, GAS skin infections that are swabbed may be those that are more prolonged and result in open infections (rather than cellulitis for example). Serology has limitations in distinguishing pharyngitis from carriage. It relies on specific cut points and timely collection of acute specimens that may not always be achieved. Some participants may have had recent GAS infections contributing to elevated antibody titres that are unrelated to their recent infection.

## Data Availability

Not applicable.

## Abbreviations

ADB: Anti-DNase B antibodies
ARF: Acute rheumatic fever
ASO: Antistreptolysin antibodies
DNA: Deoxyribonucleic acid
GAS: Group A Streptococcus
NHI: National health index
RHD: Rheumatic heart disease
WGS: Whole genome sequencing

## References

[1] Carapetis JR, McDonald M, Wilson NJ (2005). Acute rheumatic fever. Lancet.

[2] Parks T, Smeesters PR, Steer AC (2012). Streptococcal skin infection and rheumatic heart disease. Curr Opin Infect Dis.

[3] Carapetis JR, Steer AC, Mulholland EK, Weber M (2005). The global burden of group A streptococcal diseases. Lancet Infect Dis.

[4] Evidence-based, best practice New Zealand guidelines for rheumatic fever: 3. Proposed rheumatic fever primary prevention Programme. National Heart Foundation of new Zealand**,** The Cardiac Society of Australia and New Zealand [http://www.ttophs.govt.nz/vdb/document/368]. Accessed 10 Dec 2018.

[5] Nulu S, Bukhman G, Kwan GF (2017). Rheumatic heart disease: the unfinished global agenda. Cardiol Clin.

[6] Lozano R, Naghavi M, Foreman K, Lim S, Shibuya K, Aboyans V, Abraham J, Adair T, Aggarwal R, Ahn SY (2012). Global and regional mortality from 235 causes of death for 20 age groups in 1990 and 2010: a systematic analysis for the global burden of disease study 2010. Lancet.

[7] Gordis L (1985). The virtual disappearance of rheumatic fever in the United States: lessons in the rise and fall of disease. T. Duckett Jones memorial lecture. Circulation.

[8] Clemmesen S (1949). Rheumatic fever statistics in Denmark from 1878 to 1946 and their significance in profylaxis. Acta Medica Scandinavica.

[9] Milne RJ, Lennon DR, Stewart JM, Vander Hoorn S, Scuffham PA (2012). Incidence of acute rheumatic fever in New Zealand children and youth. J Paediatr Child Health.

[10] Jaine R, Baker M, Venugopal K (2008). Epidemiology of acute rheumatic fever in New Zealand 1996-2005. J Paediatr Child Health.

[11] Gurney JK, Stanley J, Baker MG, Wilson NJ, Sarfati D (2016). Estimating the risk of acute rheumatic fever in New Zealand by age, ethnicity and deprivation. Epidemiol Infect.

[12] Rheumatic Fever Report July 2017 to June 2018 [https://surv.esr.cri.nz/PDF_surveillance/RheumaticFever/Rheumaticfeverbi-annualreportJuly2017-June2018.pdf]. Accessed 10 Dec 2018.

[13] Kerdemelidis M, Lennon D, Arroll B, Peat B (2009). Guidelines for sore throat management in New Zealand. N Z Med J.

[14] More children protected from preventable diseases [http://www.beehive.govt.nz/release/more-children-protected-preventable-diseases]. Accesed 10 Dec 2018.

[15] DeMuri GP, Wald ER (2014). The group A streptococcal carrier state reviewed: still an enigma. J Pediatric Infect Dis Soc.

[16] Oliver J, Malliya Wadu E, Pierse N, Moreland NJ, Williamson DA, Baker MG (2018). Group A Streptococcus pharyngitis and pharyngeal carriage: a meta-analysis. PLoS Negl Trop Dis.

[17] Finger R, Ho SH, Ngo TT, Ritchie CD, Nguyen TN (1999). Rapid streptococcal testing in Vietnamese children with pharyngitis. Asia Pac J Public Health.

[18] Pitetti RD, Drenning SD, Wald ER (1998). Evaluation of a new rapid antigen detection kit for group A beta-hemolytic streptococci. Pediatr Emerg Care.

[19] de Silva KS, Gunatunga MW, Perera AJ, Jayamaha DJ (1998). Can group A beta haemolytic streptococcal sore throats be identified clinically?. Ceylon Med J.

[20] Upton A, Farrell E, Stewart J, Lennon D (2014). Disappointing performance of rapid antigen detection tests for group A streptococcus in the Auckland school-based sore throat programme. N Z Med J.

[21] Block SL (2014). Streptococcal pharyngitis: guidelines, treatment issues, and sequelae. Pediatr Ann.

[22] Johnson DR, Kurlan R, Leckman J, Kaplan EL (2010). The human immune response to streptococcal extracellular antigens: clinical, diagnostic, and potential pathogenetic implications. Clin Infect Dis.

[23] Danchin MH, Rogers S, Kelpie L, Selvaraj G, Curtis N, Carlin JB, Nolan TM, Carapetis JR (2007). Burden of acute sore throat and group A streptococcal pharyngitis in school-aged children and their families in Australia. Pediatrics.

[24] Esposito S, Blasi F, Bosis S, Droghetti R, Faelli N, Lastrico A, Principi N (2004). Aetiology of acute pharyngitis: the role of atypical bacteria. J Clin Microbiol.

[25] Merlini AB, Stocco CS, Schafranski MD, Arruda P, Bail L, Borges CL, Dornelles CF (2014). Prevalence of group A beta-hemolytic Streptococcus oropharyngeal colonization in children and therapeutic regimen based on antistreptolysin levels: data from a city from southern Brazil. Open Rheumatology Journal.

[26] Shulman ST, Bisno AL, Clegg HW, Gerber MA, Kaplan EL, Lee G, Martin JM, Van Beneden C (2012). Clinical practice guideline for the diagnosis and management of group A streptococcal pharyngitis: 2012 update by the Infectious Diseases Society of America. Clin Infect Dis.

[27] Kotloff KL, Dale JB (2004). Progress in group A streptococcal vaccine development. Pediatr Infect Dis J.

[28] McDonald M, Brown A, Edwards T, Hope A, Amu M, Morey F, Currie BJ, Carapetis JR (2007). Apparent contrasting rates of pharyngitis and pyoderma in regions where rheumatic heart disease is highly prevalent. Heart Lung Circ.

[29] O'Sullivan CE, Baker MG, Zhang J (2011). Increasing hospitalizations for serious skin infections in New Zealand children, 1990-2007. Epidemiol Infect.

[30] O'Sullivan L, Moreland LJ, Webb RH, Upton A, Wilson NJ. Acute rheumatic fever following group A Steptococcus pyoderma and group G Streptococcus pharyngitis. Pediatr Infect Dis J. 2017;36(7):692–4. 10.1097/INF.0000000000001558 28121967

[31] Thomas S: Risk of progression from GAS positive skin swabs to ARF. In*.*; 2019.

[32] O'Sullivan C, Baker MG (2012). Skin infections in children in a New Zealand primary care setting: exploring beneath the tip of the iceberg. N Z Med J.

[33] O'Sullivan C, Baker MG (2012). Serious skin infections in children: a review of admissions to Gisborne hospital (2006-2007). N Z Med J.

[34] Coffey PM, Ralph AP, Krause VL (2018). The role of social determinants of health in the risk and prevention of group A streptococcal infection, acute rheumatic fever and rheumatic heart disease: a systematic review. PLoS Negl Trop Dis.

[35] Jaine R, Baker M, Venugopal K (2011). Acute rheumatic fever associated with household crowding in a developed country. Pediatr Infect Dis J.

[36] New Zealand health survey 2012/13: child questionnaire (year 2). CAPI version. In field: July 2012 Update: September 2012 2012.

[37] SHIVERS [https://www.esr.cri.nz/home/about-esr/our-science-in-action/shivers-project/]. Accessed 27 May 2019.

[38] Kelly A, Denning-Kemp G, Geiringer K, Abdulhamid A, Albabtain A, Beard M, Brimble J, Campbell A, Feng S, Haminudin M et al: Housing risk factors in children admitted to Wellington Hospital. In*.* Wellington: He Kainga Oranga/Housing and Health Research Programme; 2013. 24362739

[39] Frost HR, Laho D, Sanderson-Smith ML, Licciardi P, Donath S, Curtis N, Kado J, Dale JB, Steer AC, Smeesters PR (2017). Immune cross-Opsonization within emm clusters following group A Streptococcus skin infection: broadening the scope of type-specific immunity. Clin Infect Dis.

[40] Jones S, Moreland NJ, Zancolli M, Raynes J, Loh JMS, Smeesters PR, Sriskandan S, Carapetis JR, Fraser JD, Goldblatt D (2018). Development of an opsonophagocytic killing assay for group A streptococcus. Vaccine.

[41] Bashford-Rogers RJ, Palser AL, Idris SF, Carter L, Epstein M, Callard RE, Douek DC, Vassiliou GS, Follows GA, Hubank M (2014). Capturing needles in haystacks: a comparison of B-cell receptor sequencing methods. BMC Immunol.

[42] EmMAIL - Emm Automatic Isolate Labeller [https://github.com/Andre-Tan/EmMAIL]. Accessed 10 Dec 2018.

[43] Nullarbor Github [https://github.com/tseemann/nullarbor]. Accessed 10 Dec 2018.

[44] Oliver J, Pierse N, Stefanogiannis N, Jackson C, Baker MG (2017). Acute rheumatic fever and exposure to poor housing conditions in New Zealand: a descriptive study. J Paediatr Child Health.

[45] Jack SJ, Williamson DA, Galloway Y, Pierse N, Zhang J, Oliver J, Milne RJ, Mackereth G, Jackson CM, Steer AC (2018). Primary prevention of rheumatic fever in the 21st century: evaluation of a national programme. Int J Epidemiol.

[46] Steer AC, Carapetis JR (2009). Acute rheumatic fever and rheumatic heart disease in indigenous populations. Pediatr Clin N Am.

